# Pseudoaneurysms embolization with glue via percutaneous direct puncture: a multicenter experience on 54 patients

**DOI:** 10.1186/s42155-024-00426-w

**Published:** 2024-01-15

**Authors:** Francesco Giurazza, Annamaria Ierardi, Paolo Marra, Pierleone Lucatelli, Fabio Corvino, Francesco Pane, Sandro Sironi, Gianpaolo Carrafiello, Romaric Loffroy, Raffaella Niola

**Affiliations:** 1 Vascular and Interventional Radiology Department, Cardarelli Hospital, Via A. Cardarelli 9, Naples, 80131 Italy; 2 Radiology Department, Fondazione IRCCS Cà Granda, Ospedale Maggiore Policlinico, Via F. Sforza 35, Milan, 20122 Italy; 3 Department of Radiology, ASST Papa Giovanni XXIII Hospital, University of Milano Bicocca, Piazza OMS 1, Bergamo, 24127 Italy; 4 Vascular and Interventional Radiology Unit, Department of Radiological, Oncological, and Anatomo-Pathological Sciences, Sapienza University of Rome, Roma, 00161 Italy; 5 Department of Health Sciences, Università degli Studi di Milano, Via F. Sforza 35, Milan, 20122 Italy; 6 Image-Guided Therapy Center, ICMUB Laboratory, Department of Vascular and Interventional Radiology, François-Mitterrand University Hospital, BP 77908, 14 Rue Paul Gaffarel, Dijon, 21079 France

**Keywords:** Embolization, Pseudoaneurysms, Glue, N-butyl cyanoacrylate, Percutaneous, Direct puncture

## Abstract

**Background:**

This retrospective multicentric study aims to report on technical safety and effectiveness of pseudoaneurysms embolization with glue (*N*-butyl cyanoacrylate) adopting a percutaneous direct puncture approach.

**Results:**

Fifty-four patients data were collected from five centers.

All patients at the time of treatment presented with unruptured PAs and were hemodynamically stable.

True aneurysms and lesions treated with embolics other than glue were excluded.

Pseudoaneurysms diagnosis was based on CT and anamnestic data; initial investigation with digital-subtracted arteriography was acquired in all cases; then, percutaneous embolizations were performed in the angio-suite (ultrasound, fluoroscopy, ConeBeam CT guidance) or in CT.

Technical success was considered as complete pseudoaneurysm embolization at final imaging with sole percutaneous strategy, without need for additional endovascular embolization.

Clinical success was intended as pseudoaneurysm resolution within one week follow-up with stabilization or restored clinical conditions.

Pseudoaneurysms origins were traumatic (57.4%), inflammatory (24.1%) or spontaneous (18.5%); 39 patients (72.2%) were symptomatic, presenting with pain and/or pulsatile mass.

Mean lesions diameter was 19.3 mm (range: 7–30); pseudoaneurysms were located in abdomen (48.1%), limbs (42.6%) and thorax (9.3%).

Coagulation function was impaired in 16.6% and 48.1% was under antiplatelets/anticoagulation therapy.

In 16.6% the percutaneous approach followed previous treatments failure.

The image-guidance modality for percutaneous puncture was most often ultrasound combined with fluoroscopy (38%).

Clinical success was obtained in all patients while technical success occurred in 94.4% because 3 patients required an additional endovascular embolization.

Complications were registered in 14.8%, all of low grade without clinical sequelae neither prolonged recovery (7 non target embolizations, 1 post-embolization syndrome).

**Conclusions:**

In this study, pseudoaneurysms embolization with glue via percutaneous direct puncture was safe and effective with a low rate of minor complications.

## Background

Glues are liquid embolics applied in endovascular embolizations since approximately 50 years: first cyanoacrylate application in peripheral bleeding embolizations was described by Dotter in 1975 [5].

Since then, the role of glue in the embolization field has grown and nowadays, thanks also to imaging and microcatheters developments, glues are a consolidated part of interventional radiology toolbox, especially in its endovascular use.

Seldomly, glue has been applied also via percutaneous technique, mainly in elective treatments: neck tumors [1], arterio-venous malformations [16], varices [4, 19], type-II endoleaks [18, 22] and aneurysms [11]. Regarding pseudoaneurysms (PAs) embolization, only case reports and few case-series have been published [9, 10, 21] with this approach.

This multicentric study aims now to report on PAs percutaneous embolization with glue (*N*-butyl cyanoacrylate) in a cohort of patients, analyzing safety and effectiveness.

## Methods

This is a retrospective analysis of patients with PA treated in five centers from 2020 to 2023.

Electronic radiological and medical records were reviewed.

All patients signed a written informed consent, if clinical conditions allowed. The local ethical committee approved the study.

Inclusion criteria were: PA detection at preoperative contrast-enhanced Computed Tomography (CT), anamnesis of trauma or inflammatory disease, glue embolization, percutaneous approach first, imaging follow-up almost at one week.

Exclusion criteria were: true aneurysms, additional injection of intrasaccular embolics (thrombin, cohesive liquids, coils, etc.).

PA diagnosis was based on CT findings and anamnestic data.

All patients were hemodynamically stable at the time of treatment with unruptured PAs.

Lesions features, procedural details, coagulation status and follow-up data were analyzed.

### Procedure

**Fig. 1:**
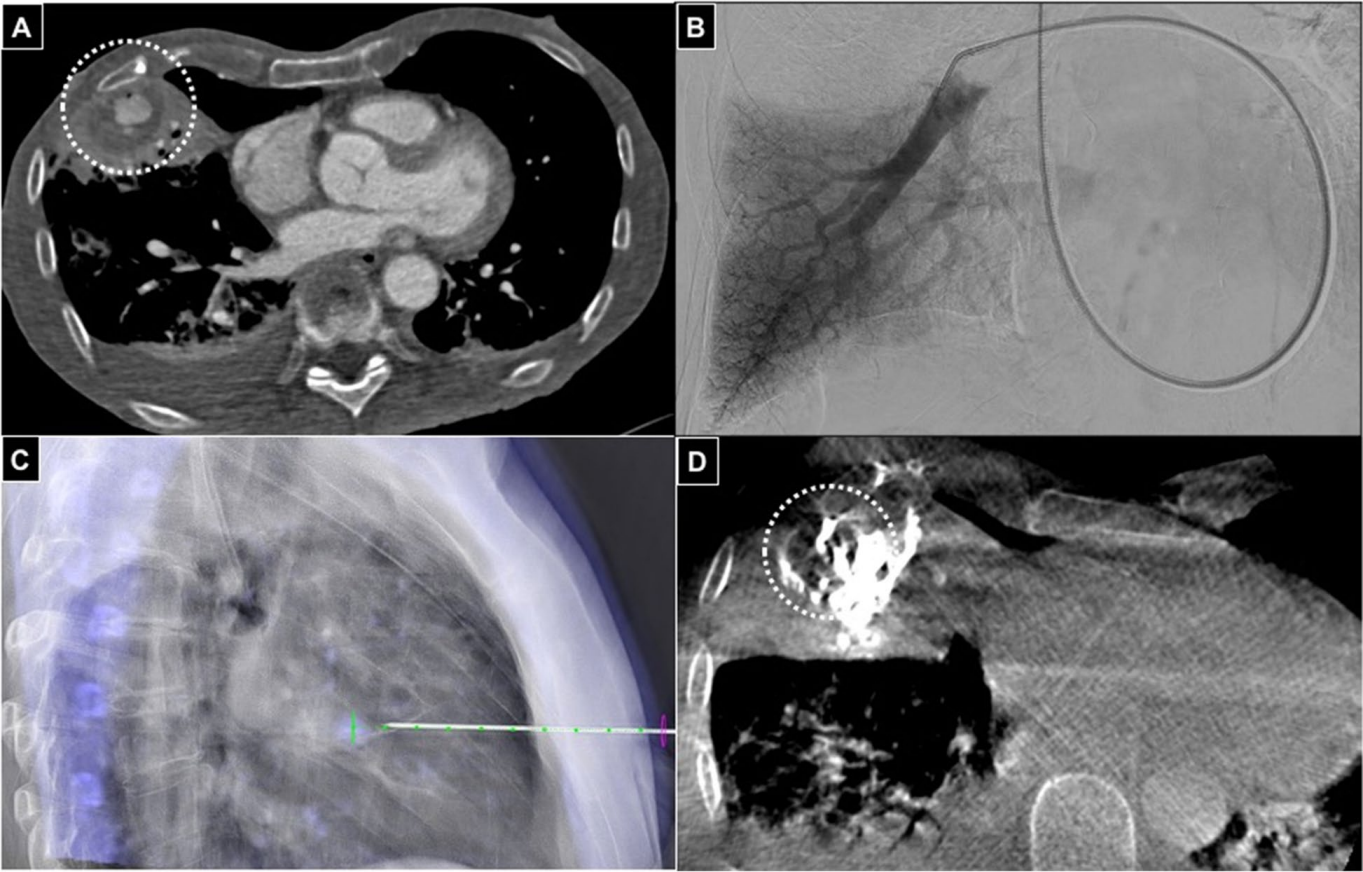
Sixty-four years old male with pneumonia. **A** Axial CT in arterial phase showing 25 mm pulmonary PA (white dotted circle) in the inferior lobe of the right lung. **B**\Right inferior lobe pulmonary DSA not depicting the lesion. **C** CBCT guided puncture using an 18G 15 cm needle with software assistance (Xpere Guide® - Philips). **D** Final CBCT showing the glue cast into the PA (white dotted circle)

**Fig. 2:**
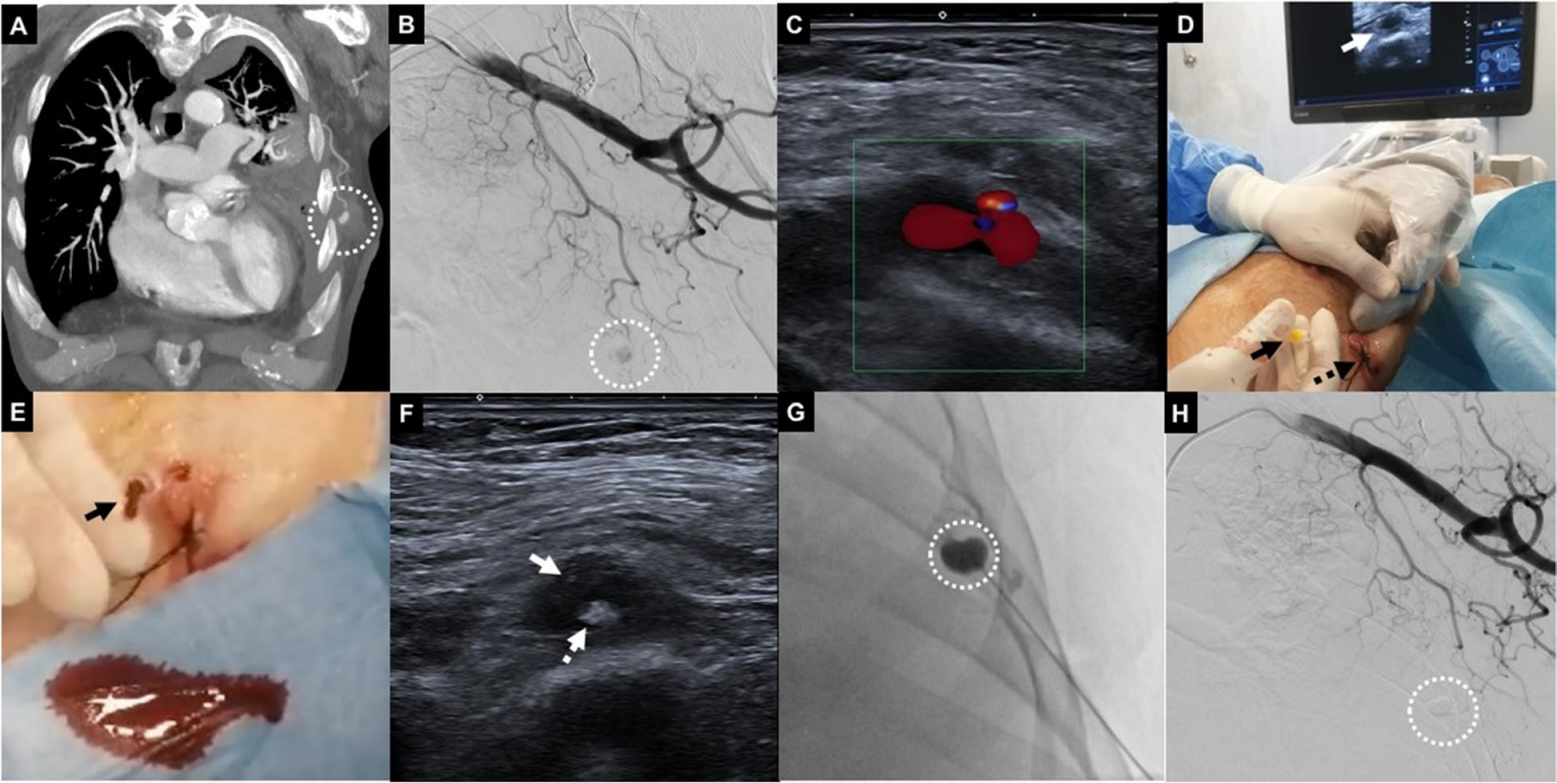
Seventy-one years old male with left thoracic wall iatrogenic bleeding after pleural drainage. **A** Coronal CT MIP reconstruction in arterial phase showing 14 mm PA (white dotted circle) refurnished by left thoraco-scapular branch. **B** Left succlavian DSA confirming the lesion (white dotted circle). **C** Color-US with linear probe identifying superficial PA. **D** Puncture of the lesion with a 20G 10 cm needle (black arrow) under US guidance (white arrow); skin suture of the previous drainage access is also evident (dotted black arrow). **E** After stylet removal, arterial blood sinked from needle hub (black arrow), confirming PA intraluminal needle tip positioning. **F** US showing proper intraluminal needle tip location (white arrow); initial glue cast formation is also evident (white dotted arrow). **G** Fluoroscopic monitoring of glue-Lipiodol injection into the PA (white dotted circle). **H** Final DSA confirming technical success, glue cast being appreciable (white dotted circle)

All interventions were performed by a single operator in the angio-suite or in CT (Figs. 1 and 2) immediately after diagnosis of PA.

After CT evaluation (Fig. 2A), a diagnostic digital-subtracted arteriography (DSA) was acquired before the percutaneous approach to confirm PA occurrence and assess lesions features in terms of morphology and vascular supply (Fig. 2B).

PAs were treated via direct puncture under image guidance (Ultrasound (US), fluoroscopy, ConeBeamCT, CT or a combination) as first choice; mandrinated needles with Quincke tip were adopted, different calipers and lengths according to operator preference and lesion site. In case of US approach (Fig. 2C-D), the needle tip could be scrubbed with a scalpel to improve its sonographic visibility [8], based on operator preference.

After local anesthesia, PA was punctured attempting to cross the largest amount of soft tissues in order to mantain the needle stable during the subsequent maneuvers; needle stylet was removed, blood gushing out from needle hub and imaging confirmed correct needle tip positioning (Fig. 2E); iodinated contrast agent was injected to opacify the PA, except in case of procedures performed under sole US guidance; then, needle lumen was flushed with glucosate 10% and finally glue (*N*-butyl cyanoacrylate, Glubran2-GEM®, Italy) mixed with Lipiodol (Guerbet®, France) was continuously injected (Fig. 2F) until PA cavity fullfilment (Fig. 2G), using slip tip syringes; needle was so retracted still injecting glue to seal the percutaneous tract and preventing eventual iatrogenic bleeding.

**Fig. 3:**
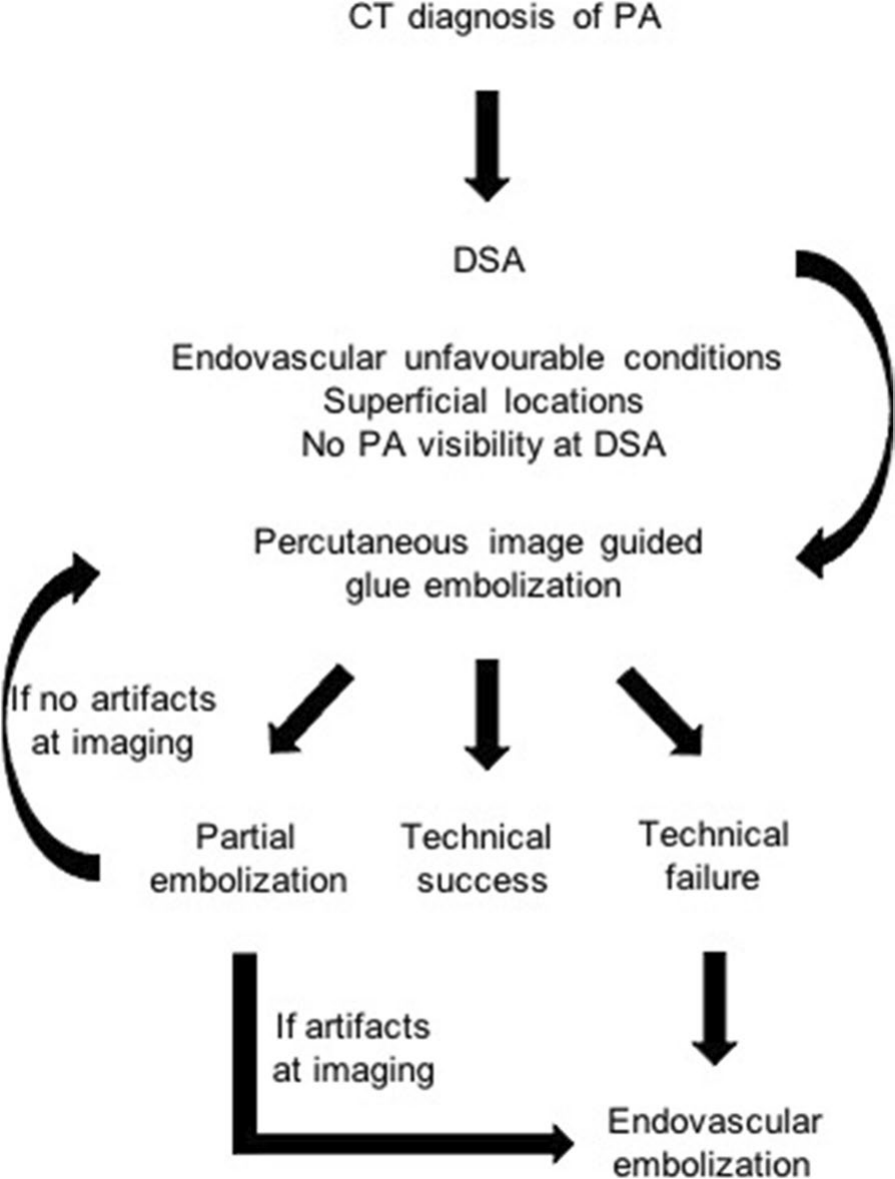
Algorithm for approaching percutaneous PA glue embolization in this study

A DSA was acquired to detect glue effects (Fig. 2H); in case of partial exclusion of the PA, other percutaneous attempts were conducted; if percutaneous puncture failed or was technically unfeasible because of impaired imaging visualization related to glue-air-Lipiodol artifacts, a standard endovascular approach was applied (Fig. 3).

The anesthesiological protocol depended on institution facilities and operators choice.

Technical success was considered as complete PA embolization at final imaging with sole percutaneous strategy, without need for additional endovascular embolization.

Clinical success was intended as PA resolution within one week follow-up with stabilization or restored clinical conditions.

Complications were evaluated according to the CIRSE classification [6].

## Results

### Sample features

The study sample included 54 patients (19 females, 35 males; mean age: 63.8 years, range 18–91) affected by PAs (Table 1).

**Table 1:** Sample features description

**Patients**	54
F	19
M	35
Mean age (yrs)	63.8
**Lesion etiology**	
Traumatic	31 (57.4%)
Inflammatory	13 (24.1%)
Spontaneous	10 (18.5%)
**Symptoms**	
Symptomatic	39 (72.2%)
Asymptomatic	15 (27.8%)
**Lesion diameter (mm)**	
Mean	19.3
Min	7
Max	30
**Lesion position**	
Abdomen	26 (48.1%)
* Visceral*	20
* Wall*	6
Limbs	23 (42.6%)
* Lower*	20
* Upper*	3
Thorax	5 (9.3%)
* Visceral*	2
* Wall*	3
**Coagulation status**	
Normal	45 (83.3%)
Impaired	9 (16.7%)
**OAC/antiPLT therapy**	26 (48.1%)

*F* Females, *M* Males, *yrs* Years, *mm* Millimeters, *OAC* Oral anticoagulant, *antiPLT* Antiplatelet

Lesions etiology was mainly traumatic, in 31 subjects (57.4%), the others being inflammatory (24.1%) or spontaneous (18.5%); 39 (72.2%) were symptomatic, presenting with pain and/or pulsatile mass.

PAs presented a mean diameter of 19.3 mm (range: 7–30) measured at preoperative CT.

Concerning lesions sites, PA were localized in abdomen (visceral: bowel 7, hepatic 5, gastric 4, splenic 2, pancreatic 1, renal 1; wall 6), thorax (visceral: peripheral pulmonary artery 2; wall 3) and limbs (lower: femoralis 10, geniculate 4, anterior tibial 3, gluteal 2, peroneal 1; upper: brachial 3).

In 9 patients (16.6%) the coagulation function was impaired: INR > 1.5 in 5, platelets < 50,000/mmc in 3 and both INR > 1.5 and platelets < 50,000/mmc in 1.

Twenty-six patients (48.1%) uptook antiplatelets/anticoagulation therapy at the time of embolization: 7 assumed oral anticoagulants (2 Fondaparinux, 3 Apixaban, 1 Warfarin, 1 Rivaroxaban), 9 heparin at prophylactic dosage, 9 antiplatelets (4 Clopidogrel, 5 acetilsalicid acid, 1 Clopidogrel plus Acetilsalicid acid), 1 heparin at prophylactic dosage and acetilsalicid acid; when the underlying pathology allowed, these therapies were suspended or shifted to heparin.

Patients with PAs caused by inflammatory and traumatic etiologies were under antibiotic regimen at the time of treatment.

### Procedural details

The procedural outcomes are summarized in Table 2.

**Table 2:** Procedural details

**Procedural rationale**	
Endovascular unfavourable	28 (51.8%)
Superficial locations	21 (38.9%)
PA not visualized at DSA	5 (9.3%)
**Previous treatments failure**	9 (16.6%)
External compression	4
Coils embolization	4
Stentgraft	1
**Glue-Lipiodol dilution**	
1:1	26 (48.1%)
1:2	18 (33.3%)
1:0.5	5 (9.3%)
1:3	5 (9.3%)
**Needle caliper (G)**	
22	17 (31.5%)
20	14 (25.9%)
18	8 (14.8%)
17	8 (14.8%)
21	7 (13%)
**Image guidance**	
US + Fluoroscopy	21 (38%)
US	16 (29.7%)
Fluoroscopy	11 (20.4%)
CT	4 (7.4%)
CBCT	2 (3.7%)
**N° of PA punctures**	
1	36 (66.7%)
2	16 (29.6%)
3	2 (3.7%)
**Anesthesiological management**	
Local	28 (51.8%)
Local + Sedation	24 (44.4%)
General	2 (3.8%)

*PA* Pseudoaneurysm, *DSA* Digital subtracted arteriography, *G* Gauge, *US* Ultrasound, *CT* Computed Tomography, *CBCT* ConeBeam CT

The rationale of adopting a percutaneous strategy, over a standard endovascular, was because of: endovascular unfavourable conditions, as tortuosity, thin collateral feeders and spasm in 28 (51.8%); operator preference related to superficial locations in 20 (38.9%); no PA detection at DSA in 5 (9.3%).

In 9 cases (16.6%) the percutaneous approach followed previous treatments failure (external compression) performed in other centers.

The glue-Lipiodol dilution most adopted was 1:1, in 26 cases (48.1%). The maximum amount of glue adopted for a single procedure was 2 vials (1 ml/vial).

Needles caliper was 22G in 17 (31.5%), 20G in 14 (25.9%), 18G in 8 (14.8%), 17G in 8 (14.8%) and 21G in 7 (13%), depending on operator preference; needle lenght varied (5 to 20 cm) according to PA location deepness.

The image-guidance modality was most often US combined with fluoroscopy in 21 cases (38%).

In 36 patients (66.7%) embolization was performed with a single puncture, in 16 (29.6%) with two punctures and in 2 (3.7%) with three.

Regarding anesthesiological management, procedures were mainly performed under sole local anesthesia (28 patients; 51.8%).

### Embolization outcome

Clinical success was obtained in all patients (100%).

Technical success occurred in 51 cases (94.4%); in 3 patients an additional endovascular embolization with glue (2) or coils (1) was needed to completely seal the PA.

Complications were registered in 8 patients (14.8%): 7 non target embolization because of glue migration and one post-embolization syndrome (PES), all without clinical sequelae neither prolonged recovery (grade I); PES required additional anti-inflammatory and anti-piretic therapy without increased prolonged hospital stay.

## Discussion

In this retrospective analysis percutaneous glue embolization of PAs was safe and effective; the most common technical approach consisted in a US-fluoroscopic guided puncture using a 22G needle and injecting a 1:1 glue-Lipiodol mixture. PAs were located in thoraco-abdominal districts, both visceral and parietal, and limbs. The lesion etiology was mainly traumatic; the rate of spontaneous lesions was also elevated compared to the general population, but this should be related to the large number of subjects uptaking antiplatelets/anticoagulation therapy in the study population.

Until now only few data are available in literature regarding this technique for the treatment of PAs. The largest series has been published by Del Corso et al. [3] on 91 patients; however all PAs were superficial and located in the limbs, mainly involving the femoral artery. Instead Carriero et al. [2] and Gorsi at al. [10] embolized abdominal visceral PAs by direct puncture using cyanoacrylate in 12 and 21 patients respectively, both obtaining 100% technical success.

Another embolic applied in percutaneous PAs embolization is thrombin; compared to glue, multiple papers have already reported its successful application, mainly in superficial districts and limbs under US guidance [12–15, 20]. Glue could so represent an alternative to thrombin: especially in case of fluoroscopy/ConeBeam CT guided procedures, where thrombin is poorly controllable, while glue mixed with Lipiodol presents the advantage of being visible [7].

Compared to the existing literature, this paper confirms safety and effectiveness of percutaneous glue PA embolization but this is one of the largest literature sample on this topic; a wide range of PA locations and etiology, as well as different image guidance techniques have been included, reflecting the everyday practice.

From a technical point of view, syringes with luer slip tip should be preferred over luer lock tip to minimize the risk of needle displacement during connection to the needle hub; samely, glue-Lipiodol mixture should be prepared in advance and ready to inject when PA is punctured. Compared to endovascular embolization, larger amounts of glue with low Lipiodol dilution and faster injection rates are adopted to fulfill directly the PA lumen, not needing glue distalization; for this reason no adjunctive procedures were performed in this sample, however some authors [17] suggested the application of endovascular balloon catheters temporary inflation close to PA neck to prevent distal migration, especially in femoral PAs percutaneous embolization.

This approach proof to be safe and effective also in patients with impaired coagulation function, glue embolic properties not being influenced; furthermore, the percutaneous tract also is sealed with glue, minimizing the risk of iatrogenic bleeding.

The complications rate was 14.8%, however it was mainly related to non target embolizations without any clinical sequela, in accordance with the technical approach: being glue injection directly performed into the target, some amount migrated to the vessels outsourcing from PA; a possible solution to reduce this event could be the application of low glue:Lipiodol dilution (1:1 or 1:0.5) to prevent unvoluntary distalization.

Regarding image guidance, US and fluoroscopy allow real time monitoring of glue injection and this is a relevant advantage compared to CT and ConeBeamCT.

Finally, even if not analyzed, this approach seems cost-effective: compared to endovascular, it would allow to reduce procedural time and costs related to microcatheters and embolics.

Main limitations of this paper are: first, the number of patients is still limited with miscellaneous PA etiology and locations, so larger and homogeneous prospective studies are needed to identify which patients will benefit the most from this approach; the technique adopted was not standardized in terms of image guidance and glue dilution because these parameters changed according to operators preference and lesions site; furthermore, a certain learning curve is required because operators should be skilled both in percutaneous interventions and glue handling; PA presented a maximum diameter of 30 mm (mean 19.3 mm), no evaluations can be provided for larger lesions; finally, follow-up is short and mid-long term results are required to verify the long-time effectiveness.

## Conclusion

In this study, PAs embolization with glue via percutaneous direct puncture was safe and effective with a low rate of minor complications.

## Data Availability

The dataset supporting the conclusions of this article is available at Cardarelli Hospital RIS-PACS system; for any questions, please contact the corresponding author.

## Abbreviations

CIRSE: Cardiovascular and Interventional Radiology Society of Europe
CT: Computed Tomography
CBCT: ConeBeam Computed Tomography
DSA: Digital subtraction angiography
G: Gauge
INR: Index Normalized Ratio
MIP: Maximum Intensity Projection
PA: Pseudoaneurysm
PES: Post-embolization syndrome
US: Ultrasound
